# Allosteric and Biased G Protein-Coupled Receptor Signaling Regulation: Potentials for New Therapeutics

**DOI:** 10.3389/fendo.2014.00068

**Published:** 2014-05-08

**Authors:** Etienne Khoury, Stéphanie Clément, Stéphane A. Laporte

**Affiliations:** ^1^Department of Medicine, McGill University Health Center Research Institute, McGill University, Montreal, QC, Canada; ^2^Department of Pharmacology and Therapeutics, McGill University Health Center Research Institute, McGill University, Montreal, QC, Canada; ^3^Department of Anatomy and Cell Biology, McGill University Health Center Research Institute, McGill University, Montreal, QC, Canada

**Keywords:** G protein-coupled receptors, allosterism, biased signaling, functional selectivity, receptor domains

## Abstract

G protein-coupled receptors (GPCRs) are seven-transmembrane proteins that participate in many aspects of the endocrine function and are important targets for drug development. They transduce signals mainly, but not exclusively, via hetero-trimeric G proteins, leading to a diversity of intracellular signaling cascades. Ligands binding at the hormone orthosteric sites of receptors have been classified as agonists, antagonists, and/or inverse agonists based on their ability to mainly modulate G protein signaling. Accumulating evidence also indicates that such ligands, alone or in combination with other ones such as those acting outside the orthosteric hormone binding sites (e.g., allosteric modulators), have the ability to selectively engage subsets of signaling responses as compared to the natural endogenous ligands. Such modes of functioning have been variously referred to as “functional selectivity” or “ligand-biased signaling.” In this review, we provide an overview of the current knowledge regarding GPCR-biased signaling and their functional regulation with a focus on the evolving concept that receptor domains can also be targeted to allosterically bias signaling, and discuss the usefulness of such modes of regulation for the design of more efficient therapeutics.

## Introduction

G protein-coupled receptors (GPCRs) constitute the largest family of cell-surface receptors and are involved in almost all physiological and hormonal responses. Therefore, it is not surprising that they are the most targeted in drug discovery programs (1). Their activation was first described by a classical two-state model, where receptors exist in equilibrium between active (e.g., G protein-coupled: the “on” state) and inactive states (e.g., G protein-uncoupled: the “off” state), and where extracellular stimuli, such as hormones, neurotransmitters, peptides, and amino acids, shift this equilibrium from one state to the other. Based on this model, the properties of ligands were classified as agonists, antagonists, and inverse agonists, according to their ability to stabilize the “on” state of receptors allowing the full activation of G proteins such as for agonists, reducing the basal spontaneous coupling to G proteins like in the case of the inverse agonists (e.g., maintaining receptor in the “off” state), or inhibiting agonist competitively without changing the equilibrium like for “neutral” antagonists. To explain the biological and physiological responses triggered by these ligands, this binary model also assumes that GPCRs preferentially couple to one G protein subtype, and that either agonists, antagonists, or inverse agonists affect in a similar manner – according to its respective class – the activation of such G protein. Moreover, if more than one G protein subtype binds to its cognate receptor, each class of ligands would also affect it in a similar manner. However, several new lines of evidence now support an alternative multi-state model, where GPCRs can adopt multiple conformations, including active, inactive, and other intermediate ones. In such multi-state model, it is also inferred that ligands have the propensity of stabilizing a unique conformation leading to a specific signaling response, which may or may not always totally mimic the one induced by a natural ligand of reference. These ligands can stabilize a “hybrid” receptor conformation that mimics the “on” conformation with respect to engaging one signaling pathway, while at the same time mimicking the “off” conformation for another signaling pathway that is normally activated by an agonist of reference. Such mode of ligand-mediated differential signaling is commonly referred to as “functional selectivity” or “ligand-biased signaling,” and would in principle allow the activation of specific pathways and cell responses. It is now well-accepted that many orthosteric ligands (OL) have the ability to bias signaling between different G proteins and/or between G proteins and β-arrestins that are involved in the desensitization, internalization, and signaling of GPCRs. This latter mode of biased signaling has already been extensively covered in many recent reviews (2–4), and will not be furthermore expanded here.

Functional selectivity is not only limited to OLs, but is also a property that has been described for other allosteric ligands (AL)/effectors. These, which are also known as “allosteric modulators,” include ions, ligands, small and large molecules (e.g., antibodies) and/or protein complexes (e.g., receptor dimers and receptor–effector complexes) that modulate hormone binding and/or the intracellular coupling of receptors to their effectors, and affect responses in different ways: they can have differential cooperative effects – negative or positive ones – on the binding of the OL, the “conduit” of the information of the ligand to the effector through the receptor, and the biasing of receptor signaling. Moreover, an evolving concept also suggests that receptor domains that participate in ligand and/or effector binding, and in the “signaling conformations” of GPCRs, can also be targeted to bias signaling. Here, we will expand on this concept of targeting receptors’ domains to allosterically regulate their signaling for mainly class A and C GPCR, and review some of the potential clinical and pre-clinical uses of biased AL.

## Biasing GPCR Signaling by Allosteric Modulators

**Figure 1:**
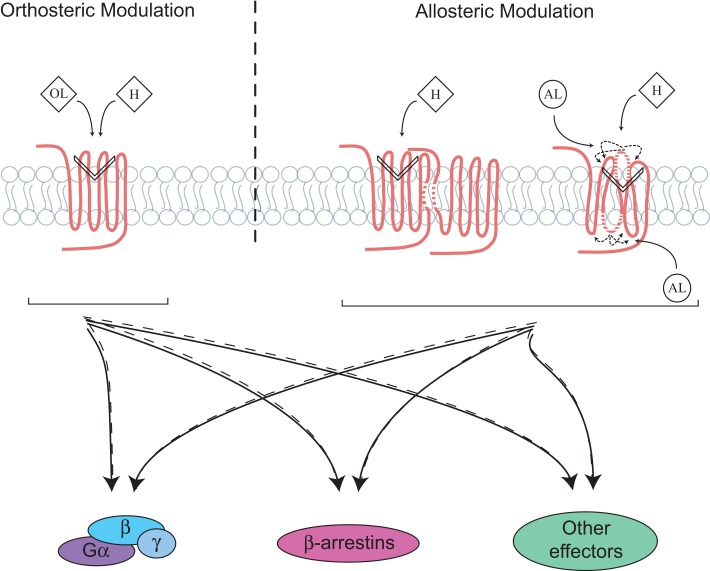
**Directing signaling by orthosteric and allosteric GPCR ligands, receptor complexes and domains**. Signaling occurs following the binding of endogenous hormones (H) to the orthosteric site on G protein-coupled receptor (GPCR), which involves different domains, mainly the transmembrane and extracellular ones. This leads to the activation of multiple signaling pathways that are balanced between the G proteins, β-arrestins, and/or other signaling effectors. Ligand-directed signaling (i.e., biased signaling) can occur through the binding of either orthosteric (OL) or allosteric (AL) ligands to the receptor, which changes the balance of signaling between the effectors as compared to a ligand of reference. Formation of receptors complexes, such as dimers as well as reorientation of extra/intracellular domains of receptors can also lead to conformational rearrangements or be targeted for biased signaling.

Allosterism was first described with the hemoglobin, where the binding of oxygen to a specific site increases the affinity of other oxygen molecules to the remaining unoccupied sites (5). Over the years, this concept has proven to be widely spread for various types of proteins, and more recently for transmembrane receptors, such as GPCRs. Interestingly, numerous endogenous allosteric modulators have been identified, and shown to play crucial roles in keeping diverse biological functions mediated by this class of receptors. To date, the best characterized GPCR allosteric modulator is the G protein itself, which binds receptors and stabilizes their active conformation. Moreover, ions, such as Zn^2+^, Na^+^, and Ca^2+^ are other examples of endogenous molecules that have been shown to allosterically modulate GPCRs (6). In addition, other types of endogenous modulators, such as the small tripeptide Pro–Leu–Gly (PLG), also known as the melanocyte-stimulating hormone release inhibiting factor (MIF-1) has also been shown to act as an allosteric modulator on the D_2_ and the D_4_ dopamine receptors. PLG increases the affinity of dopamine for its receptors and the agonist-mediated inhibition of adenylyl cyclase (AC) (7). Similarly to OLs that have the capacity to bias GPCR signaling, AL, which bind to topographically distinct sites from the endogenous ligands, can direct receptor signaling (Figure 1). They can either exert positive, negative, or neutral effects on receptor signaling, and these modulatory effects do not always parallel those seen on the binding of OLs. Specifically, AL can influence the binding of the orthosteric hormone to its receptor, which can be independent from its impact on the signaling transduction promoted by the OL itself. Their effects on receptor signaling have been mainly divided into two categories: positive allosteric modulators (PAMs) and negative allosteric modulators (NAMs). Many advantages are known to be associated with the use of AL in terms of fine tuning GPCR responses (8, 9). First, because AL bind sites on GPCRs that are more diverse in nature than the orthosteric ones, greater selectivity can be achieved with such ligands. Indeed, targeting specific receptors belonging to the same family subtype has been often challenging because of their highly conserved sequences and structures within their orthosteric binding sites. For instance, class C GPCRs, group II metabotropic glutamate receptors (mGluRs), including mGluR2 and mGluR3, were selectively targeted by a NAM, which only inhibited the glutamate-induced response of group II receptors without exerting any effect on groups I and III mGluRs (10). Second, the effect of allosteric modulators on receptors is also saturable, since they are not competing with endogenous ligands. In other words, when all allosteric sites are occupied on receptors, no more effects are achieved. Third, most allosteric modulators are known to exert their function only in the presence of the endogenous ligand. Indeed, the AL will modulate the receptors’ conformation and signaling only when the endogenous hormone occupies its orthosteric site. However, some AL have also been described to act as allosteric agonists and promote functional effects in the absence of OLs and those are known as ago-allosteric modulators. Such ligands have also been referred to as “super-agonists,” because they can act synergistically with the natural ligand. An example of ago-AL is the phenylacetamide 1 and 2, which act directly on the free fatty acid receptor 2 (FFA2) (11).

Another property of AL is also their ability to bias receptor signaling. Such allosteric modulation has been mainly studied in class A and C GPCRs. For example, a recent study characterized a new compound, PDC113.824, acting as an allosteric modulator on the class A GPCR, the prostaglandin F2α (FP) receptor. This compound was shown to act as a PAM on the agonist-mediated Gαq signaling pathway, while at the same time acting as a NAM on the Gα12 signaling cascade (12). Another example of G protein-dependent biased signaling is revealed by an autoantibody, which targets a class C GPCR, the calcium-sensing receptor (CaSR) and allosterically modulates the calcium-mediated potentiation of Gαq signaling, while inhibiting the Gαi response (13). Moreover, the gadolinium (Gd^3+^), a known allosteric modulator of the mGluR1α, was shown to act as a PAM on the agonist-mediated Gαq and a NAM on the Gαs signaling pathway (14). Allosteric-biased modulation on GPCRs is not only limited to G proteins vs. G proteins signaling but can also occur between G proteins and other signaling effectors, such as β-arrestins. For instance, the ATI-2341 compound acting on the C–X–C chemokine receptor type 4 (CXCR4) was recently shown to display differential ago-allosteric properties between G protein and β-arrestins signaling (15). ATI-2341 preferentially promoted the coupling of CXCR4 to Gαi- over Gα13-mediated signaling, but contrarily to the endogenous ligand, did not induce β-arrestin recruitment to the receptor. On the other hand, a study on the cannabinoid receptor 1 (CB1) showed that the ORG27569 compound acted as an NAM on the agonist-mediated Gαi signaling and a PAM on β-arrestin-dependent internalization of CB1; and acted as an ago-allosteric modulator on both receptor internalization and β-arrestin-dependent MAPK signaling (16, 17). These examples highlight the diversity of GPCR allosteric modulators on both receptors and their ensuing signaling (e.g., PAM and NAM effects). However, defining how they differentially direct downstream effectors and responses still remains an empirical endeavor for most receptors. Moreover, because allosteric modulators have the potential to differentially affect distinct effectors and responses, much more work still remains to better survey all the potential signaling pathways that can be affected, even the ones that are not normally suspected to be activated by the receptor studied.

## Targeting GPCR Domains for Allosterically Biasing Receptor Signaling

Because different domains of GPCRs [e.g., intracellular loops (ICL) and extracellular loops (ECL) and transmembrane domains (TM)] are known to participate in ligand and/or effector recognition and receptor dimerization, recent attention has also been drawn to understand the role of these domains in receptor conformation and signaling. It is well-recognized that GPCRs can form oligomers within which conformational rearrangements of the receptors can impact signaling (Figure 1) (18, 19). An example is the heterodimerization of the class A GPCRs chemokine receptors, CXCR4 and CXCR7 (19). This study showed that, when receptors are both in complex, agonist-mediated activation of Gαi is impaired, whereas β-arrestin is constitutively recruited to the dimer. Because other reviews have already covered the concept of GPCR oligomers and its ensuing effects on directed signaling, such topic will not be furthermore discussed here (20–22). Of note, however, is a seminal study on the dimerization of the β2-adrenergic receptor (β_2_AR), which used a peptide consisting of residues 276–296 of the TM6 of the receptor (GIIMGTFTLCWLPFFIVNIVH), to prevent homodimerization, showed that the agonist-mediated cAMP production could be inhibited; thus suggesting that domains of GPCRs could be targeted to regulate receptors signaling in an allosteric fashion (23). In addition, another study has also demonstrated that peptides derived from TM of CXCR4 and CCR5 could be used as specific receptor antagonists (24). Despite that TM are involved in ligand binding and receptor activation, targeting these regions with peptides has proven to be difficult due to their hydrophobic nature.

Receptor ICLs are critical for GPCR signaling and can also be targeted to modulate receptor’s responses (Table 1). For instance, studies using mimics of ICL3 from many different GPCRs, such as the adrenergic receptors α_1B_ and α_2A_ (α_1B_AR and α_2A_AR), as well as the muscarinic acetylcholine receptors M_1_ and M_2_ (M_1_AChR and M_2_AChR), have revealed the importance of this domain in G protein coupling/activation (25). These peptides, which are derived from regions of receptors’ loops were shown to disrupt both Gαq and Gαi coupling to their cognate receptors and to affect downstream signaling. Similar effects were also reported for the angiotensin II type-2 receptor (AT_2_R) (26). Moreover, because β-arrestins also bind ICLs, peptides derived from the ICL3 and ICL1 of receptors were also shown to block GPCR densensitization (27). For example, a synthetic peptide corresponding to the sequence of the full length of ICL3 of the luteinizing hormone/choriogonadotropin receptor (LH/CGR) was shown to reverse the agonist-mediated desensitization of AC activity when incubated with membranes expressing LH/CGR by preventing the interaction between β-arrestin and the receptor (28). Not only was targeting receptor’s ICL shown to affect G protein and β-arrestin binding, but it was also demonstrated to bias GPCR signaling between these different pathways. Indeed, the ago-allosteric ATI-2341 compound, which belongs to the pepducin family and that is a short lipidated peptide of the ICL1 of the CXCR4, was shown to promote biased signaling between G proteins and β-arrestins (15). Consistent with the idea that intracellular domain of GPCRs can be targeted to allosterically bias receptor signaling, a recent study on the β_2_AR, using different antibodies directed against its intracellular domains, showed that the recruitment of β-arrestin to the receptor and the activation of G proteins were differentially affected (29). Targeting GPCR’s ICLs with peptide mimics has proven efficient for regulating receptor signaling. However, such approach can suffer from the impediment of having to modifying the peptides (e.g., lipidation) for their cellular delivery. On the other hand, the modification of peptides with lipids would favor reaching higher plasma concentrations of peptide near its target, the receptor, which would presumably increase their activity as compared to a gene delivery approach.

**Table 1:** **Role of GPCRs intracellular (ICL) and extracellular (ECL) domains in receptors function**.

Domains	Receptors	Functions	Reference
ICL	C–X–C chemokine receptor type 4	A pepducin derived from ICL1 acts as allosteric agonist	Quoyer et al. (15)
	β2-adrenergic receptor	Intrabodies targeting ICLs act as allosteric ligands	Staus et al. (29)
	C–C chemokine receptor type 3	Different residues of ICLs are important for agonist-induced cellular responses (orthosteric)	Auger et al. (30)
	Dopamine D2 and D3 receptors (D2R–D3R)	Certain residues in ICL2 are important for the agonist-induced translocation of arrestin3 (orthosteric)	Lan et al. (31)
	α2-adrenergic receptor (α2AR)	Certain residues in ICL3 are important for agonist-induced signaling (orthosteric)	Small et al. (32)
	Rhodopsin receptor	Mimics of the ICL3 and ICL1 allosterically blocked arrestin binding	Krupnick et al. (27)
	Luteinizing hormone/choriogonadotropin receptor (LHCGR)	Mimic of the ICL3 allosterically blocked arrestin-dependent desensitization	Mukherjee et al. (28)
ECL	V1α vasopressin receptor (V1αR)	Residues of ECL2 are important for agonist binding and receptor activation (orthosteric)	Conner et al. (33)
	V2 vasopressin receptor (V2R)	ECL1–2 mimics act as bias, allosteric ligands	Rihakova et al. (34)
	M3 muscarinic acetylcholine receptor (m3AChR)	Residues of ECL2 are important for agonist-mediated signaling (orthosteric)	Scarselli et al. (35)
	Somatostatin receptor (SSTR)	Anti-ECL2 antibodies act as selective allosteric agonists	Leu and Nandi (36)
	Prostaglandin F2α receptor (FP)	ECL2 mimic acts as an allosteric modulator	Peri et al. (37), Goupil et al. (12)
	C–C chemokine receptor type 5 (CCR5)	Anti-ECL2 antibodies allosterically block HIV entry	Blanpain et al. (38)
	C–C chemokine receptor type 5 (CCR5)	ECL2 mimic, acting as an allosteric modulator, blocks HIV entry	Dogo-Isonagie et al. (39), Thathiah et al. (40)
	Parathyroid hormone 1 receptor (PTH1R)	Different residues of ECL3 are important for PTH (1–34) binding (orthosteric)	Lee et al. (41)
	Adenosine A2B receptor (A2BR)	Different residues of ECL1 are important for agonist-mediated receptor activation (orthosteric)	Peeters et al. (42)

The ECLs of GPCRs are also important in ligand binding and receptor activation (Table 1). Using site-directed mutagenesis, residues in the ECL2 [Phe(189), Trp(206), Phe(209), and Tyr(218)] of the V1α vasopressin receptor, which are highly conserved amongst GPCRs, were shown to be critical for receptor activation (33). Another study focusing on the parathyroid hormone 1 receptor (PTHR) used a similar approach and identified residues in the ECL3 (Trp-437 and Gln-440), which were important for PTH (1–34) binding (41). Mutagenesis studies of the adenosine A2B receptor have also implicated residues of the ECL1 in receptor activation (42). The ECL2 was also shown to be important for agonist binding and the activation of the M3 muscarinic acetylcholine receptor (M_3_AChR) (35), the C–C chemokine receptor type 5 (CCR5) (43), the dopamine D2 receptor (D2R) (44), and the complement factor 5α receptor (C5αR) (45). Other studies have also explored the contribution of ECL2 in stabilizing receptor conformations. For example, structural studies using NMR spectroscopy reveal that the ECL2 forms a cap that stabilizes the inactive conformation of the receptor and changes its orientation upon receptor activation (46).

The ECL domains of GPCR can also be targeted to allosterically modulate signaling. For instance, a recent study screened potential small molecule for their action on the relaxin/insulin-like family peptide receptor 1 (RXFP1), with the goal of finding agonists. They identified compound 8, which displayed agonistic effects on cellular responses such as cAMP production and cellular impedance. Interestingly, using different RXFP1 constructs and mutagenesis, they showed that the ECL3 of the receptor was required for this effect, suggesting that the compound interacts with an allosteric site within this ECL (47). Conversely, another study showed that the agonist effects of the allosteric modulators (phenylacetamide 1 and 2) on the FFA2 receptor were lost when its ECL2 was replaced with one of the FFA3 subtype receptor (48). Moreover, antibodies against the ECL2 have been shown to modulate receptor activity. In the case of the somatostatin receptors (SSTR), antibodies directed against their ECL2 have agonistic-like properties, while having no effect on agonist binding to receptors. SSTR2, SSTR3, and SSTR5 selective antibodies were also shown to diminish cAMP production and decrease serotonin secretion leading to a suppressed proliferation of neuroendocrine tumor cells (36). Moreover, specific antibodies against the ECL2 of the CCR5 were shown to block HIV entry in cells (38). Similarly, a peptide mimic of the C-terminal portion of CCR5 was shown to act as a NAM for HIV-1 entry (39). Not surprisingly, because ECLs can adopt different conformations in GPCR, either at basal state or upon receptor activation (46, 49), it can also be targeted to allosterically bias receptor signaling. For instance, a study on the vasopressin V2 receptor (V2R) using peptides corresponding to a decapeptide of ECL1 (PPLLARAELA) or an octapeptide of ECL2’s C-terminus (ALCRAVKY) showed physiological functional selectivity on different vasopressin-mediated responses in a non-competitive manner (34). Moreover, a peptide derived from a sequence overlapping the N-terminus of ECL2 and the TM4 of the prostaglandin F2α (FP) receptor, known as THG113 (ILGHRDYK), was able to block PGF2α-mediated contraction of the myometrium in a non-competitive manner (37). Supportive for the allosteric regulation of the FP receptor signaling, is the finding that a peptide mimic of THG113, the PDC113.824 compound, was also able to induce functional selectivity on G protein signaling mediated by PGF2α (12). Also consistent with the idea that the ECL2 can be targeted to direct receptor signaling is the use of an allosteric modulator of the M2 and M4 muscarinic acetylcholine receptors, LY2033298 compound, that was shown to bias downstream signaling (50). Interestingly, a congener of the LY2033298, the LY2119620 compound was later shown, through co-crystallographic studies, to interact with residues of the ECL2, and to allosterically alter M2 receptor active conformation (51). The GPCRs’ ECLs represent promising targets for allosterically modulating receptors, as they are presumably more accessible than ICLs. In particular, ECL2 is a good target, because of its diversity amongst GPCRs and because it is involved in the binding of OLs and in the signaling of many receptors. However, much more needs to be understood about how ECLs regulate ligand binding, receptor conformation, and signaling, and how peptides derived from these regions affect these functions.

## Therapeutic Potentials of Allosteric and Biased Signaling

Recently, great attention has been devoted to functional selectivity as a new paradigm applicable for the development of better therapeutic drugs with potentially fewer off-target and/or side effects. An example is highlighted with the use of the biased agonist pilocarpine, which selectively acts on the M1 muscarinic acetylcholine receptor and shows positive therapeutic effects in different Alzheimer’s disease models. Specifically, pilocarpine biased Gαq-mediated phospholipase C activation over the Gαs-mediated AC stimulation, whereas the non-selective muscarinic agonist carbachol equally stimulated responses mediated by Gαs and Gαq (52, 53). Moreover, biasing β-arrestin-dependent signaling has also been shown to be potentially beneficial in heart diseases. For example, TRV120027 (Sar–Arg–Val–Tyr–Ile–His–Pro-d-Ala-OH), a peptide antagonist of the AT1R-dependent Gαq pathway was recently shown to selectively induce β-arrestin signaling. TRV120027 increases cardiomyocyte contractility *in vitro*, and cardiac performance both in rats and dogs (54, 55), suggesting that this orthosteric-biased ligand could be beneficial in acute heart failure treatment (54). Another example is the biased ligand TRV130, which acts on the μ-opioid receptor and induces cAMP inhibition through a Gαi-dependent mechanism without inducing either β-arrestin recruitment or receptor internalization. This biased agonist, which has similar potency and efficacy on Gαi signaling as morphine, showed higher analgesic efficacy, lower respiratory suppression, and less gastrointestinal dysfunction when compared to morphine (56).

Because AL can bias GPCR signaling, such modulators also represent interesting opportunities for drug discovery (Table 2). Moreover, greater subtype selectivity amongst receptors can be achieved using AL, which may also improve therapeutic benefits. For instance, the ADX10059 compound, a selective NAM for the mGluR5, was shown to improve symptoms in patients suffering from gastro-esophageal reflux disease (GERD) (57, 58). On the other hand, Reparixin (formerly Repertaxin), which acts as a NAM on both chemokine receptors, CXCR1 and CXCR2, shows promising therapeutic effects on the prevention of delayed graft dysfunction after kidney transplantation and early stages of breast cancer (59, 60). Research efforts focusing on targeting the ECL2 of GPCRs to allosterically modulate signaling, have also led to the development of new drugs. Maraviroc, a peptide that was originally developed from a mimic of the ECL2 of CCR5 (discussed in previous section), decreases the viral load in HIV-1 patients (61). Because of the putative role of CCR5 in alloreactivity, Maraviroc is also in phase II clinical trial for acute graft-versus-host disease (GVHD). Moreover, the ECL2 mimic of the FP receptor, PDC31 (derived from the THG113 peptide), which was shown to inhibit preterm labor in different animal models, is now being evaluated in a clinical phase II trial for primary dysmenorrhea (37, 62). Other examples of allosteric and biased drugs are currently under pre-clinical investigation or in clinical trials (Table 2).

**Table 2:** **Potential therapeutic usage of allosteric and biased GPCR signaling compounds/drugs**.

Receptors	Drugs	Indications	Reference
Calcium-sensing receptor (CaSR)	Cinacalcet (marketed)	Hyperparathyroidism	Goodman et al. (63)
C–C chemokine receptor type 5 (CCR5)	Maraviroc (marketed)	AIDS/HIV	Fätkenheuer et al. (61)
C–X–C chemokine receptor type 1/2 (CXCR1/2)	Reparixin	Reperfusion injury in lung and kidney transplantation	Bertini et al. (59), Zarbock et al. (64)
Prostaglandin F receptor (FP)	PDC31 (THG113.3)	Preterm labor and primary dysmenorrheal	Olson and Ammann (62); (http://clinicaltrials.gov/show/NCT01250587)
Metabotropic glutamate receptor 2 (mGluR2)	ADX71149	Schizophrenia	Hashimoto et al. (65)
Metabotropic glutamate receptor 2/3 (mGluR2/3)	AZD8529	Schizophrenia	(http://clinicaltrialsfeeds.org/clinical-trials/show/NCT00985933)
Metabotropic glutamate receptor 5 (mGluR5)	AFQ056	Parkinson’s disease levodopa-induced dyskinesia and fragile X syndrome	Berg et al. (66), Jacquemont et al. (67)
	Dipraglurant (ADX48621)	Parkinson’s disease levodopa-induced dyskinesia and dystonia	Stocchi et al. (68); (http://www.addextherapeutics.com/rd/pipeline/dipra-ir/)
	ADX10059	Gastro-esophageal reflux	Zerbib et al. (57); Stocchi et al. (68)
	RO4917523	Depression and fragile X	http://clinicaltrials.gov/ct2/show/study/NCT01517698
	Fenobam	Fragile X	Berry-Kravis et al. (69)
	STX107	Fragile X and autism	http://clinicaltrials.gov/show/NCT01325740

## Conclusion and Perspectives

The possibility of directing GPCR signaling with either OL or AL has opened new opportunities for developing more selective/effective drugs; because, in principal, it would allow a better control of the signaling pathway(s) involved in the underlying pathophysiology, hence limiting side effects that may be engendered from the non-selective engagement of other GPCR subtypes and/or from unwanted downstream signaling effectors/responses. However, much more is needed to be understood about directed signaling in order to develop better therapeutics; especially about GPCR interaction with biased ligands (orthosteric and allosteric ones) and their ensuing effects on receptor conformation, and how such receptor–ligand complex can convey signaling information in a selective manner. Challenges also include a better understanding of the role played by GPCR domains such as the TMs, ECLs, and ICLs in the conformation of receptors and how different OL and AL ligands affect these domains to stabilize subsets of conformations for selective signaling. In particular, because allosteric compounds may represent a promising new class of drugs, predicting their sites of binding on GPCRs and modes of action – on the hormone binding, conformation, and “conduit” of signaling (e.g., ago-agonist, PAM, or NAM) – becomes fundamental. Moreover, because most GPCR can engage many downstream signaling pathways, which in many cases are cell-, tissue-, and/or context-specific, it is essential to better define the entire “signaling repertoire” for the hormones/drugs of reference and the putative-biased ligands to be studied, particularly, in normal vs. pathological conditions. Establishing such “signaling repertoire” becomes therefore essential in programs that aim at improving drug efficacy or at repurposing drugs; hence, future research efforts should be oriented toward the development of approaches for assessing the full spectrum of signaling of the ligands of interest, in different cell models, including those physiological and pathophysiological relevant ones, and integrative analytical methods for linking these “signaling signatures” to pre-clinical and/or existing clinical data.

## Conflict of Interest Statement

The authors declare that the research was conducted in the absence of any commercial or financial relationships that could be construed as a potential conflict of interest.
